# Passive Cigarette Smoking Impact on Blood Pressure Response to Epinephrine and Felypressin in 1K1C Hypertensive Rats Treated or not with Atenolol

**DOI:** 10.36660/abc.20180433

**Published:** 2020-02

**Authors:** Camila A. Fleury, Elizandra P. M. Almeida, Thiago J. Dionisio, Adriana M. Calvo, Gabriela M. Oliveira, Sandra L. Amaral, Carlos F. Santos, Flávio A. C. Faria

**Affiliations:** 1 Faculdade de Odontologia de Bauru - Universidade de São Paulo - Ciências Biológicas - Farmacologia, Bauru, SP - Brazil; 2 Programa Conjunto de Pós-Graduação em Ciências Fisiológicas - Departamento de Educação Física - Faculdade de Ciências - Universidade Estadual de São Paulo, Bauru, SP - Brazil

**Keywords:** Tobacco Use Disorder, Hypertension, Rats, Felypressin, Atenolol, Epinephrine, Tobacco Smoke Pollution, Nicotine/adverse effects

## Abstract

**Background:**

Cigarette smoking is usually associated with hypertension and may modify vasoconstrictor response.

**Objective:**

The present study aimed to analyze and compare the interaction of passive cigarette smoking and hypertension on epinephrine and felypressin blood pressure effects after intravascular injection.

**Method:**

45-day male Wistar rats had the main left renal artery partially constricted and the right kidney removed (1K1C model). Rats were placed in the chamber for exposition to passive cigarette smoking (10 cigarettes) during 10 min (6 days a week). Hypertensive rats received atenolol (90 mg/kg/day) by gavage for two weeks. Hypotensive and hypertensive response, response duration and heart rate were recorded from direct blood pressure values. The significance level was 5%.

**Results:**

Passive cigarette smoking increased maximal hypertensive response to epinephrine in normotensive and 1K1C-atenolol treated rats and to felypressin only in 1K1C-atenolol treated rats; it also reduced epinephrine hypotensive response. Epinephrine increased heart rate in normotensive and hypertensive passive smokers or non-smoker rats. Comparing the two vasoconstrictors, epinephrine showed greater hypertensive response in normotensive smokers, 1K1C-atenolol treated smokers and non-smokers. However, in normotensive-nonsmoker rats, felypressin showed a greater and longer hypertensive effect.

**Conclusions:**

Our results suggest that passive cigarette smoking may reduce epinephrine vasodilation and increase hypertensive response when compared to felypressin. Therefore, felypressin may be safe for hypertensive patients to avoid tachycardia and atenolol interaction, but for normotensive and non-smoker patients, epinephrine may be safer than felypressin.

Introduction

Vasoconstrictor drugs are essential for dental and medical procedures performed under local anesthesia, since the local anesthetic must stay in contact with sensitive nerves. One single local anesthetic cartridge administered via intravascular route can be fatal.^1^ Therefore, vasoconstrictors are also used to avoid its absorption and adverse effects: seizures, arrhythmia and cardiac arrest. There are studies indicating vasoconstrictor absorption and systemic effects. Epinephrine can be detected in plasma after infiltration and can increase heart rate in normal subjects.^2^ There are no clinical data comparing epinephrine and felypressin efficacy or safety, but there is evidence that isolated local anesthetics results in shorter and low quality pain control.^3^

Vasoconstrictor drugs have systemic effects that can be critical in patients with cardiovascular diseases: coronary vasoconstriction, tachycardia, increases in cardiac contraction force, etc. Such effects are related to the most common causes of death in the modern world: heart attack, stroke and thrombosis; and patients usually have multifactorial diseases. Cigarette smoking and hypertension are included in the National Cholesterol Education Program algorithm to predict cardiovascular disease risk, addressing both as modifiable causes of atherosclerosis in prevention efforts.^4^ The WHO estimates that the global prevalence of adults smoking any tobacco product is 36% in adult men and 8% in adult women.^5^

The number of cigarettes show a positive correlation with higher blood pressure values but not with heart rate.^6^ Nicotine also affects the autonomous nervous system, having a different effect than cigarette smoking products.^7^ Catecholamine release and atherosclerotic lipoproteins are increased in the blood levels of smoker patients.^8^ The vasoactive drugs present, therefore, alter the vascular response of smokers, since the nitric oxide (NO) pathway, adrenergic and cholinergic systems are affected. Although epinephrine is considered the safest vasoconstrictor drug in patients with cardiovascular disease by The American Dental Association,^9^ patients receiving betablocker treatment who smoke cigarettes must be treated differently.

Felypressin may be a safe alternative vasoconstrictor in such populationce since it is a non-adrenergic vasoconstrictor. There is no reported interaction between cigaretes smoking and vasopressin. A previous study showed reduced hypertensive effect of felypressin in rats treated with atenolol.^10^ In order to increase the knowledge about the safety of epinephrine and felypressin, this study attempted to test them isolated in intravascular administration to simulate the maximal error in local anesthesia, providing information for the formulation of safer local anesthetic solutions. Therefore, the aim of this study was to evaluate and compare the effects of direct intravenous injection of epinephrine and felypressin on blood pressure of smoker or non-smoker normotensive, hypertensive and atenolol-treated hypertensive rats.

## Methods

For the present study, all norms for animal research were reviewed and approved by the institutional review board before the experiments (protocol #010/2010). Male Wistar rats weighing 140 to 320 g provided by *Faculdade de Odontologia de Bauru* facilities were used in all groups of this study. The experimental hypertension method, indirect and direct BP measurements, were performed as previously described^10^. All rats received a normal diet, free water and food access and were submitted to 12 hour light/dark cycle. Rats weighing 110 to 150 g were anesthetized with an injection of ketamine (50 mg/kg weight, im, Dopalen^®^- Sespo Industry and Trade Ltda., Animal Health Vetbrands Division - Jacareí, São Paulo, Brazil) plus xylazine (10 mg/kg weight, im, Anasedan^®^- Sespo). The left renal artery was isolated, and a 0.25 mm gap silver clip was installed around it, and the right kidney was completely removed. 40,000 IU of small-animal antibiotic (Fontoura Wyeth S.A. - São Bernardo do Campo, São Paulo, Brazil) was injected. It is worth mentioning that surgery for clips implantation in the renal arteries was performed in 10 animals per group, that is, 120 rats. However, each groups consisted of 6 animals, since part of the rats did not have systolic blood pressure above 150 mmHg. Therefore, 72 was the total number of animals used in the study and there were no criteria for the definition of this sample, being defined by convenience.

### Passive Smoking Method

Passive smoking was performed based on previous emphysema induction studies^11^. One day after hypertension induction, the rats were intoxicated one time a day (10 cigarettes per exposure period), 6 days a week. The exposure protocol consisted of confining 10 rats in the inhalation chamber during 10 min of compressed air ventilation (10 L/min).

### Indirect and Direct Blood Pressure Measurements

1K1C rats were heated in individual cages containing an electrical resistance and tail pneumatic cuffs were installed and connected to a digital system for indirect blood pressure record (Physiological Pressure Transducer, ADInstruments Pty. Ltd. - Dunedin, Otago, New Zealand). Rats that presented systolic blood pressure equal to or higher than 150 mmHg in the indirect measurement 15 days after clip surgery were accepted in the hypertensive group or treated with atenolol (90 mg/kg/day; Cristália Pharmaceutical and Chemical Products - Itapira, São Paulo, Brazil) administered by gavage in 1mL for 2 weeks.

**Figure 1:**
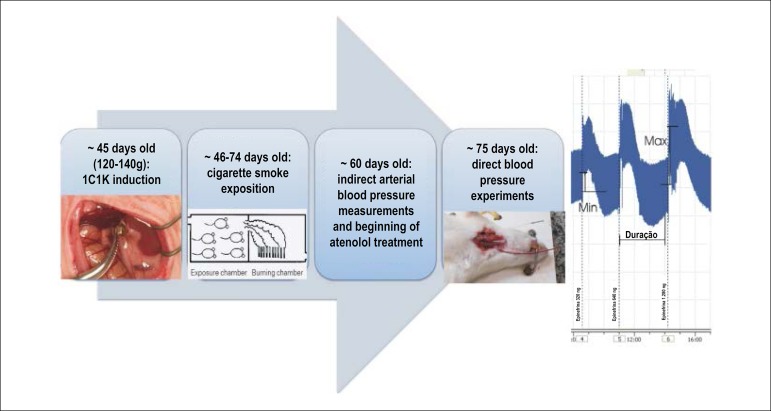
Schematic study protocol: at 45 days of life, the rats were submitted to clip implantation (1K1C hypertension induction); on the next day, the smoker groups were exposed to cigarette smoke; at 60 days of life, blood pressure was measured by indirect means and atenolol treatment was started; approximately at 75 days of life, direct blood pressure experiments were performed. An example of pulsatile recording after epinephrine administration details how parameters were calculated.

All groups had blood pressure measured directly for 28-35 days after clip surgery, or the equivalent time in the control group: after ketamine/xylazine anesthesia, a saline-filled polyethylene catheter PE-50 (Clay Adams - Franklin Lakes, New Jersey, USA) with an occluded external extremity was implanted in the left carotid artery and in the right jugular vein. The arterial catheter was connected to a pressure transducer coupled to an invasive blood pressure recording system, using appropriate software (Physiological Pressure Transducer; PowerLab 4/30; Chart Pro - ADInstruments Pty. Ltd). The experiments of intravenous injection of vasoconstrictor drugs were performed with anesthetized rats right after catheter implantation. A scheme of the experiments design are detailed in Figure 1.

### Dose-Response Curves to Epinephrine and Felypressin

Exogenous epinephrine (Adren^®^- HipolaborFarmacêuticaLtda - Belo Horizonte, Minas Gerais, Brazil) diluted in saline solution was injected at the doses of 80, 160, 320, 640 and 1,280 ng in bolus through tin venous catheter to obtain dose-response curves. Felypressin alone (Dentsply Pharmaceutical, Catanduva, São Paulo, Brazil) was used at doses of 0.125, 0.25, 0.5, 1, 2 and 3mIU. Intravenous injections in random order were performed after a 3-min interval for each response to stabilize blood pressure. Animals were euthanized with intravenous injection of excessive doses of the anesthetic drug thiopental (Thiopentax^®^, Cristália - Chemical and Pharmaceutical Products). The following parameters were analyzed using PAM values (PAM = 1/3 Systolic Pressure + 2/3 Diastolic Pressure): minimal hypotensive response, maximal hypertensive response and response duration. Duration was determined using global pressure alterations, since epinephrine has a complex blood pressure response where there is a hypertensive peak followed by hypotensive response and normalization; previous studies have clarified this pattern^10^. Heart rate was recorded 30 s after the injection during one min to avoid bias caused by longer duration or great blood pressure changes since the program used pulsatile pressure to determine these parameter. In order to compare epinephrine and felypressin, doses that corresponded to 1, 2, 4 and 8 local anesthetic cartridges which would have been administered to the rats, were used. The following formula was used: D = 18,000 (for epinephrine*) or 54 mIU (for felypressin*) x 4,286 x 10^-3^ (a weight correction from humans (70 kg) to rats (300 g).

### Statistics

For all groups, n = 6. The results were expressed as Mean ± Standard Deviation (SD) for data with normal distribution according to Kolmogorov-Smirnov test. In cases where normal distribution did not occur in one group or more, data were presented as Median ± interquartile interval.

When more than one response was obtained from the same animal, repeatd -measures one-way analysis of variance was used (Repeaed- measures One-Way ANOVA). To compare complete curves from two or more groups, repeaed- measures Two-Way analysis of variance was used (Repeaed- measures Two-Way ANOVA). When there was a significant difference between the doses in each curve or in independent groups and normal distribution, comparison was performed by Holm-Sidak’s or Tukeyhodtest. For non-parametric results, Mann-Whitney and Kruskal-Wallis tests were used. The level of significance in this study was set at 5% (p < 0.05). All tests were performed using STATISTICA Software (StatSoft South America).

## Results

Basal blood pressure values during the first five minutes are summarized in Table 1 and used as the initial reference. Atenolol significantly reduced blood pressure in hypertensive animals, whose values were still significantly higher than in control animals.

**Table 1:** Basal values

		Weight (g)	Mean BP (mmHg)	Systolic BP (mmHg)	Diastolic BP (mmHg)	Heart rate (BPM)
Non-smoker	Normotensive	285.00 ± 25.09	112.7 (101.7-118.8)	140.5 (123.2-144.0)	99.6 (93.1-103.4)	210.5 (194.1-225.1)
	1K1C Hypertensive	297.83 ± 30.20	163.1 (155.3-177.7)^a^	201.1 (188.3-231.9)^a^	135.2 (127.9-148.6)^a^	205.9 (196.2-248.9)
	1K1C Atenolol-Treated	215.00 ± 34.29^a^	148.9 (136.1-163.4)^a.b^	184.9 (175.3-201.2)^a.b^	132.5 (119.3-142.8)^a.b^	185.7 (176.5-207.7)
Smoker	Normotensive	302.50 ± 19.04	104.1 (93.6-112.4)	134.8 (119.2-145.2)	86.2 (81.6-95.8)	225.1 (206.2-249.4)
	1K1C Hypertensive	250.58 ± 16.21^c^	156.6 (152.7-160.3)^a^	194.35 (188.8-203.3)^a^	129.7 (126.5-145.3)^a^	219.7 (207.6-231.1)
	1K1C Atenolol-Treated	254.42 ± 21.86^c^	126.2 (117.7-138.1)^a.b^	153.5 (148.6-167.5)^a.b^	106.1 (102.8-111.1)^a.b^	212.6 (200.0-218.7)

Values of weight; basal values for mean, systolic, diastolic arterial pressure and heart rate obtained during the first five minutes for smoker and non-smoker normotensive, 1K1C hypertensive and 1K1C atenolol-treated groups (n = 12). Weight - Mean ± Standard Deviation. Arterial Pressure and Heart Rate - Median (25^th^ Percentile-75^th^ Percentile).

a p < 0.05 compared with normotensive group.

b p < 0.05 compared with hypertensive group

c p < 0.05 compared with non-smoker respective group.

### Maximal Hypertensive Response

**Figure 2:**
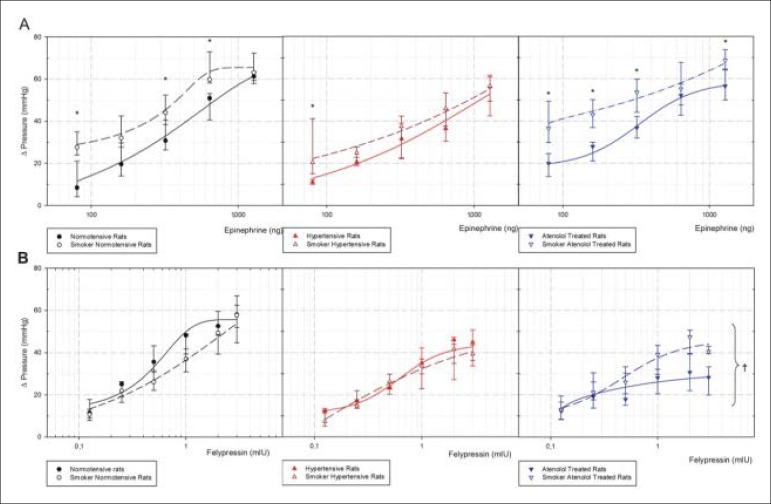
Maximal hypertensive response curves after intravenous injection in bolus of epinephrine (A) or felypressin (B) in control non-smoker normotensive and smoker normotensive rats, non-smoker 1K1C hypertensive and smoker 1K1C hypertensive, non-smoker 1K1C atenolol-treated and smoker 1K1C atenolol-treated rats. n = 6 for all groups. Median (25^th^ Percentile-75^th^ Percentile). *p < 0.05 vs non-smokers groups.

Maximal hypertensive response curves for epinephrine and felypressin, smokers vs. non-smokers, are shown in Figure 2. Smoking significantly increased epinephrine maximal hypertensive responses in normotensive and atenolol-treated rats. Smoke significantly increased felypressin maximal hypertensive response only in the atenolol-treated group.

### Minimal Hypotensive Response

**Figure 3:**
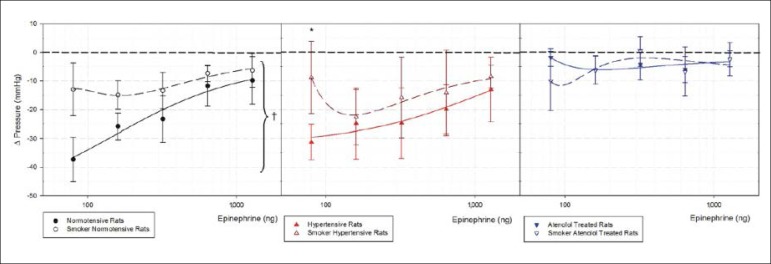
Minimal hypotensive response curves after intravenous injection in bolus of epinephrine in control non-smoker normotensive and smoker normotensive rats, non-smoker 1K1C hypertensive and smoker 1K1C hypertensive, non-smoker 1K1C atenolol-treated and smoker 1K1C atenolol treated rats. n = 6. Mean ± Standard Deviation. *p < 0.05 vs non-smokers groups.

Figure 3 shows epinephrine minimal hypotensive response curves. Smoke significantly reduced the hypotensive response in normotensive rats (p < 0.05). There was a significant reduction in vasodilator response in 1K1C atenolol-treated group after epinephrine administration. Felypressin, as expected, did now result in significant hypotensive response in the three studied groups (Figure 3B).

### Heart Rate

**Figure 4:**
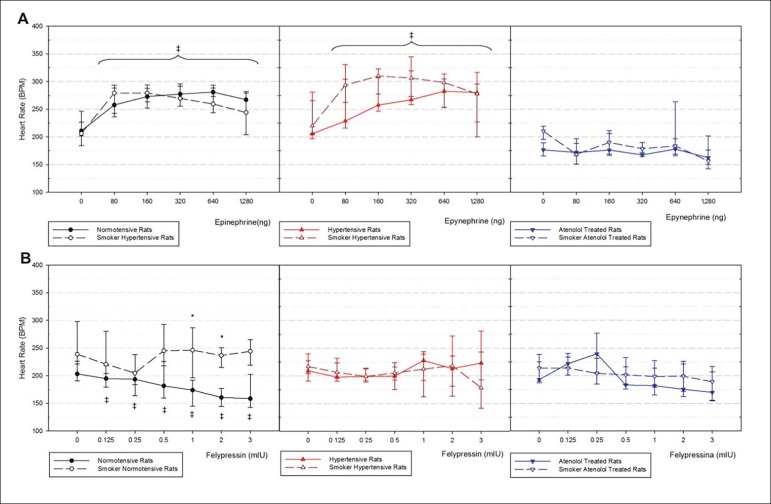
Heart rate before and after intravenous injection in bolus of epinephrine (A) and felypressin (B) in control non-smoker normotensive and smoker normotensive rats, non-smoker 1K1C hypertensive and smoker 1K1C hypertensive, non-smoker 1K1C atenolol-treated and smoker 1K1C atenolol-treated rats. n = 6 for all groups. Median (25^th^ Percentile-75^th^ Percentile). *p < 0.05 vs non-smokers groups. ‡ p < 0.05 compared to basal values.

Epinephried caused a significant increase in heart rate in normotensive and 1K1C hypertensive rats when compared with basal values for each group, but smoking did not alter such effect (Figure 4A). 1K1C atenolol-treated rats showed no changes in this parameter, probably due to the antagonistic effect of atenolol on b_1_-receptors. Felypressin showed a significant reduction in heart rate for non-smoker, normotensive rats when compared with basal values (Figure 4B). Smoke significantly increased heart rate only at 1 and 2 mIU doses of felypressin, when compared with non-smoker normotensive control rats.

### Response Duration

Response duration is described in Table 2. Felypressin showed a significantly longer duration of blood pressure responses than epinephrine in all studied groups.

**Table 2:** Response Duration

Number of cartridges	Normotensive				Hypertensive				Atenolol-treated rats			
	Nonsmoker		Smoker		Nonsmoker		Smoker		Nonsmoker		Smoker	
	Epinephrine	Felypressin	Epinephrine	Felypressin	Epinephrine	Felypressin	Epinephrine	Felypressin	Epinephrine	Felypressin	Epinephrine	Felypressin
1	453.5 (412-517)	905.2 (748,6-945)*	276.5 (214-298)	908 (880-948)*	297 (278-333)	911.5 (880-949)*	325.5 (277-492)	818.5 (610-876)*	291.5 (260-352)	811 (754-914)*	426 (382-470)	692.5 (552-738)*
2	427 (399-471)	1,042 (928.2-1118.9)*	268.5 (256-302)	888.5 (796-947)*	421 (376-436)	979.5 (934-1012)*	344 (329-497)	875.5 (771-972)*	398 (345-444)	545 (496-708)*	498 (413-616)	960 (920-976)*
4	445 (378-466)	1,117 (1,050-1,184.4)*	287 (294-323)	1,004.5 (876-1,086)*	422.5 (351-464)	1,021 (920-1,080)*	475 (357-513)	954.5 (882-1,042)*	415.5 (380-453)	712 (552-858)*	421 (405-464)	938 (783-1,111)*
8	455 (423-506)	1,077.1 (983-1,193)*	353 (332-365)	1,066.5 (1,000-1,170)*	455 (425-538)	1,032.5 (1,011-1,229)*	472.5 (400-507)	1,087 (998-1,154)*	454 (393-489)	946 (800-1,103)*	511.5 (479-586)	1,063.5 (987-1,240)*

Response duration (in seconds) after intravascular administration of epinephrine or felypressin (dose contained in the corresponding number of local anesthetic cartridges) in smoker and non-smoker normotensive, 1K1C hypertensive and 1K1C atenolol-treated groups (n = 6 for all groups). Median (25^th^ Percentile-75^th^ Percentile).

* p < 0.05 compared with epinephrine.

### Comparison between Epinephrine and Felypressin

**Figure 5:**
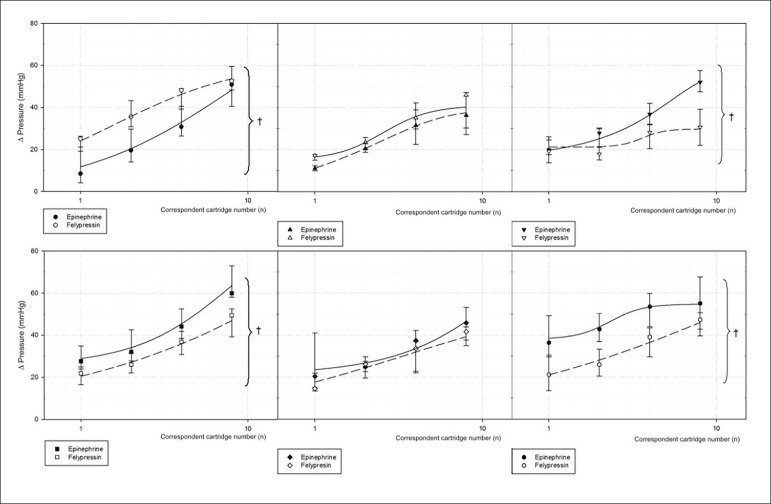
Comparison of maximal hypertensive response curves after intravenous injection in bolus of epinephrine and felypressin in control non-smoker normotensive, smoker normotensive rats, non-smoker 1K1C hypertensive, smoker 1K1C hypertensive, non-smoker 1K1C atenolol-treated and smoker 1K1C atenolol-treated rats. n = 6 for all groups. Median (25^th^ Percentile-75^th^ Percentile). *p < 0.05 in the comparison between drugs.

When comparing felypressin to epinephrine responses in each group, felypressin showed a reduced hypertensive effect on smoker normotensive, smoker 1K1C-atenolol treated and non-smoker 1KC -atenolol-treated rats (Figure 5). In smoker and non-smoker hypertensive rats, there was no significant difference between both vasoconstrictors. In non-smoker normotensive rats, felypressin resulted in a greater hypertensive effect when compared with epinephrine.

## Discussion

We associated smoke with hypertension and atenolol treatment in an attempt to reproduce a multifactorial disease. In order to provide safety information about isolated use of vasoconstrictors via intravascular route, which cannot be tested in humans, our study aimed to test if epinephrine is contraindicated in the smoker and hypertensive population and to provide a safe alternative through the analyses of systemic effects. Epinephrine was used as the vasoconstrictor of choice for hypertensive patients according to the American Heart Association (AHA,^9^, compared to felypressin, less often studied and not associated with the sympathetic autonomous nervous system^12^.

Our data show that epinephrine maintained the same blood pressure responses, either in hypertensive smoker or hypertensive nonsmoker rats without atenolol treatment (Figures 2A and 3), but significantly increased heart rate (Figure 4A). However, in atenolol-treated non-smoker rats, the blood pressure response was higher compared with smokers. Considering all data about epinephrine safety in combination with local anesthetic use in the hypertensive population^13^, our results suggest that the hypertensive response to epinephrine in hypertensive smoker and non-smoker rats without atenolol treatment, remains unchanged. On the other hand, the hypertensive, smoker and atenolol-treated rats showed significantly higher blood pressure measurements when compared with non-treated animals.

It is still not clear if cigaretes smoking increases blood pressure values, as some studies indicted that it can potentiate family history and increase systolic, diastolic and mean arterial pressure value.^6^. In our study, the smokers groups did not show increased basal blood pressure values when compared with non-smokers (Table 1). Smoke exposure was carried out for 4 weeks, as expected, and this time was sufficient to alter vasoconstrictor response. Rats were submitted to an epinephrine or felypressin intravascular injection under ketamine/xylazine anesthesia; it was shown in a previous study by our group that such mixture reduced basal heart rate but did not alter blood pressure responses^10^.

Smoke and nicotine can act diversely; nicotine seems to reduce blood pressure when administered acutely, while cigaretes smoking products are associated with increased blood pressure value.^7^. Tobacco smoke sidestream reduces acetylcholine endothelium-dependent relaxation when compared to non-smokers.^14^ Our data show that epinephrine-induced hypertensive responses were increased in normotensive and atenolol-treated hypertensive smoker rats when compared to the non-smokers groups (Figure 2) and when compared to felypressin (Figure 5). Blood pressure is defined by cardiac output multiplied by vascular peripheral resistance. Felypressin injected by the intravenous route shows only a vasoconstrictor effect, increasing vascular resistance and leading to an increase in blood pressure values. Epinephrine, on the other hand shows vasoconstrictor and vasodilator, cardiac inotropic and chronotropic effects, leading to complex blood pressure responses after an intravenous injection. Vasodilation reduces hypertensive responses when global blood pressure is measured in and normotensive non-smoker rats showed the highest values of hypotensive response for the lowest doses of epinephrine (Figure 3), reducing hypertensive response when compared with felypressin (Figure 5). Felypressin presents less cardiac effects, but significantly reduced heart rate on normotensive non-smoker rats; such results are consistent with previous studies, where this vasoconstrictor response was associated with prilocaine,^15^ which was associated with coronary artery constriction and baroreflex.

There is a complex cardiovascular response to nicotine because of ganglionar nicotine receptors which influence autonomous sympathetic and parasympathetic nervous systems. This response includes increase in catecholamine release and altered lipids metabolism which explains increase in cardiovascular disease development on in smokes.^8^. According to the Third Report of National Cholesterol Education Program (NCEP), smoking cigarettes has a direct impact on atherosclerosis formation and increases cardiovascular disases risk.^4^ The association of hypertension and cigarettes smoking represents a delicate case for vasoconstrictor use. Felypressin response duration was significantly higher than epinephrine’s in all groups, which was expected since vasopressin half-life is approximately 17-35min,^16^ while epinephrine has a short half-life due to metabolism and synaptic reuptake.

Cigarette smoking increases blood vessel stiffness by different pathways, including oxidative stress increase of endothelin-1 production and formation of smooth muscle cell.^17^. Nicotine-free cigarette smoke extract administered by subcutaneous injection induced endothelial dysfunction, increased blood pressure values and reduced acetylcholine-induced vasodilatation.^18^ Although the chemical component responsible for epithelial dysfunction is not clear, cigarettes smoke extract reduces vascular relaxation by increasing oxidative stress and reducing NO bioavailability.^19^ Smokers also showed altered lipoprotein metabolism, increased levels of oxidized low density lipoprotein (LDL) which may contribute to vasoconstriction.

Chronic smoking impairs NO synthesis and enhances production of reactive oxygen species, while nicotine administration leads to hypertension due to increased sympathetic nervous system.^2,21^ Our study showed a significantly reduced minimal hypotensive response on the smoker normotensive group (Figure 3). This reduced vasodilation caused by epinephrine may be related to the increase on the smooth muscle layer promoted by smoke. The heart rate effect was similar in both smoker and non-smoker groups.

## Conclusion

Epinephrine and felypressin dosage corresponded to the content of 0.5 to 16 local anesthetic cartridges administered *in bolus* via intravascular route in hypertensive rats but there were no deaths. Our results support vasoconstrictor safety in associated vascular problems, mostly especially felypressin seems to be promising vasoconstrictor to smoker patients since there is no interaction with the sympathetic nervous system.
